# Chicago and the Cholera

**Published:** 1884-08

**Authors:** 


### CHICAGO AND THE CHOLERA.

When a stranger visits the city of Chicago for the first time, his earliest request is to be shown the "Water-Works and the Stock-Yards." These are the features for which the town has established a reputation. People who come to it from afar, from the Orient and the Occident, ask to see these wonders for which the Mistress of the great Lake is famous. Some cities have a reputation for ancient statuary; some, for marvelous paintings; some, for musical culture; some, for rare pottery; some, even for *pate de foie gras!* But it is not for any of these that Chicago has become famous. It is for her Stock-yards and her Water-works.

We understand that at this time there is unwonted activity in and about the municipality, among editors, physicians, sanitarians, members of health-boards, and private citizens, on the subject of the possibility of an invasion of the city by the cholera scourge. In view of this wide-spread apprehension, and the precautionary measures resulting in the form of alley-cleaning, tenement-house inspection, etc., it becomes a duty as well as a pleasure to assure all who are interested, that Chicago will not and can not suffer from cholera. The basis of such a positive assertion is, as a matter of fact, to be found in the two things for which the city is famous. It is because of the Water-works and the Stock-yards.

The sacred singer of Israel once cried in a burst of poetic imagery, "Moab is my wash-pot!" Chicago might well echo with the refrain, "Lake Michigan is my wash-pot, my drinking-cup, and my chamber-pot!"

Her Water-works are indeed great. They supply her with water from the lake fully and freely, in fact, as fully and as freely as she restores to it again the refuse of her corporate body. If there be any germ of disease in her excreta, any virus lurking in her animal refuse, she receives it again in part, after it has been thrown off. If there be any potency in high dilutions, any truth in the doctrines of *similia similibus*, Chicago is safe from the cholera! If there be any virtue in the attenuation of a virus, such as has been demonstrated in the now famous experiments on rabies and fowl cholera, Chicago is, without a shadow of a doubt, secure! It is the Water-works to which she is indebted for this priceless immunity. Her position is this: "I put my trust, not in disinfection, not in combustion, not in antisepsis, not in agents capable of destroying bacteria, but in two miles of lake water! That is the safe limit! Whether agitated by a boisterous storm, or as placid as a mountain-girt basin, these two miles shall stretch between me and all my sanitary sins! I will micturate, defecate, eject excreta, at one end of these two miles, and drink my water at the other, and I shall be safe! No wonder they come to see my Water-works! With these shall I not be cholera-proof!"

And there are those also who visit her Stock-yards! Not content with emptying her own ejecta into the lake from which she drinks, Chicago pours into it also the drainings from her immense cattle-yards, and from the great slaughter-houses where the animals brought to her doors from the whole of the North-west are cut up and packed for the consumption of the world. The following is a description of the results of this great sanitary movement, which we clip from one of the daily journals, and which, from observation, we can declare to be within the bounds of sober truth:

"The South Fork is that fork of the South Branch of the Chicago River starting at Thirty-ninth and Halsted streets and emptying into the South Branch in Bridgeport near the pumping-works. It is about three miles in length and is the filthiest of all the branches of the river. The foulest, filthiest, deadliest section lies just north of the Union Stock-yards, and is about one mile in length, from 100 to 200 feet in width, and from nine to eighteen feet deep. It is wholly unprotected as to shade, there being no trees nor houses on either side, and when the sun shines upon it, it seethes and boils like a great cauldron of oil. To many intelligent people an inspection of the waters of this inland body would be a revelaation. The dark, seething mass lying between the two banks of what was once a respectable creek cannot be called water. It is not refuse in its ordinary state. If it were white instead of black it might be taken for yeast. Its consistency in some quarters is about the same as paint, and when it comes in contact with the hull of a vessel it leaves a coating usually about an inch in thickness which has become known to the marine classes as 'Stock-yards paint.' Something of its character may be judged from the fact that water will not wash it off, and that the only way it can be removed is by taking the vessel into the dry-docks and using a steel scraper. Unlike the ordinary paint, it does not serve to prolong the use of timber, but, on the contrary, serves to hasten its decay.

"No living thing can drink this water and survive. It is a poison even to the grass that grows along the shore if poured upon it in large quantities. When used sparingly and mixed with other materials it serves as a fertilizer equal to bone phosphates and guano. Seen at a distance the surface of the water looks white, but a careful examination discloses that this is due to the bubbles which fill the scum, and that both it and the liquid are really as dark as stove-polish. The scum varies in thickness, depending upon the weather. The sun brings it up, and the wind sometimes drives it toward the pumping-works, two miles distant. A small quantity of it is sometimes carried away by the works, but much of it is again wafted back.

"At times this frothy portion, which is of the consistency of dough, forms a layer on the surface of the water two feet thick. Generally it is mixed with dead rats, dead dogs, dead cats, pieces of flesh, blood, and hair. An enterprising man has erected a lot of sheds near one corner of the Stock-yards, where he conducts a double-barreled business. Certain seasons, when the weather is not too warm, he gathers in acres of the scum, which is carried through a certain process by which it is relieved of the fatty substances. This has proven itself of great value to soapmakers, to whom several car-loads are sold each season. When the sun is too hot for this work, the soap-grease man turns his attention to the fertilizer business. Large quantities of the filth is pumped into vats on the shore and evaporated. It is then sold to a large firm which mixes it with other materials and sells it for a fertilizer. When reduced and weakened by a mixture of bone dust it makes one of the richest improvers of worn-out soil yet discovered, but if used in its ordinary strength it would burn up everything planted near it.

"Many people suppose the South Fork is relieved by the Bridgeport Pumping-works, but the good the works do amounts to nothing at this point. The fork acts as a drainage for all large packing-houses in the vicinity of the Stock-yards. How feeble the effect of the pumping-works is may be appreciated when the fact is stated that the current of the fork, if a weary, inch-by-inch movement may be described by the use of that word, is in the opposite direction, toward a sewer, the opening of which is near Thirty-ninth and Halsted streets, and running towards the lake. Taking the statements of intelligent people whose interest in the locality have led them to study the subject, it is safe to say that this accumulation of rottenness never loses its character altogether, no matter what the season may be, and a history of the present mass would go back many years into the past.

"The effect of the stench depends upon the wind. In other words, it is naturally strongest in the face of the wind, and the people who live round about for a mile each way greet the changes of the weather-cock with alternate joy and sorrow. It is treated as a veritable river of death. On walking out in that direction the visitor unacquainted with the ground begins to feel that something strange and startling is about to happen, or has already happened. The situation is all the more difficult to comprehend if the wind happens to be blowing toward the river. Then it is difficult to comprehend why people going and coming hurry forward with such serious countenances. With many it has become a habit to quicken their footsteps as they approach the place, and they do so whether the wind comes that way or not, and the same pace is kept up on the other side until the place is cleared. Even when the odor is for a time suppressed the air for miles around seems heavy and wanting in those elements which give ease and pleasure to the processes of respiration."

The witches' cauldron here correctly described, exhales its pestilential breath about two miles from the spot where some of the wealthy "packers" of this city have erected palaces which a king might envy, their costly stones overlooking the fashionable thoroughfare, where youth, beauty, premature age, and folly drive their showy equipages in a splendid ignorance of what is floating beyond! More than half a million men, women, and children have breathed in attenuation the vapors of this pestilential pool, and consumed the water, which in high dilution contains the power and potency of its multiplying germs. How idle to worry over any importation from Toulon, Marseilles, or other city of France, when Chicago has even more than can be imported, right at her own door! Shall she lament over the introduction of a few paltry rags from Egypt, when she has a greater rottenness in the grasp of her right hand! If the south fork of the Chicago river be not capable of destroying thousands where an imported pestilence could but kill its hundreds, it would not be part and parcel of this astonishing metropolis! No! Chicago will not have the cholera! She is protected by "vaccination" from a great deal worse! She has her wash-pot, her chamber-pot, and her drinking-cup in the great lake at her feet, and can look with a smile into the rheumy eyes of the Filth-Plague!

It is, however, true that Chicago *has* suffered from the cholera. But that was in an older time! Years after the scourge that once afflicted her had vanished, the boatmen on the river could see the relics of its grim repast in the ends of the rude slab coffins that projected from the sand-banks on either side the stream. But that was long before half a million of human beings and many more animals had converted the river into an open sewer! They had no Water-works and Stock-yards in those primitive days! Then there was no "vaccination"-process for protection. The untutored hunter of scalps and his friend, the Indian trader, then had their little stools down stream, and drank from the water above. Being thus quite unprotected, they fell easy victims to the foreign germ!

But to-day, as the dark shadow of the scourge falls across the Atlantic, it is so much lighter in effect than the south fork of the Chicago river, that the spot is even brightened by the reflection! Chicago has her Water-works and her Stock-yards! She is safe!

(Sanitary and other exchanges are welcome to copy!)

